# Anti-CV2/Collapsin Response Mediator Protein 5 (CRMP5) Paraneoplastic Encephalitis Induced by Small Cell Lung Cancer

**DOI:** 10.7759/cureus.34323

**Published:** 2023-01-29

**Authors:** Veigar Þór Helgason, Pir Abdul Ahad Aziz Qureshi, Vikram Rao Bollineni, Björn Logi Þórarinsson, Enrico B Arkink

**Affiliations:** 1 Department of Radiology, Landspítali - The National University Hospital of Iceland, Reykjavík, ISL; 2 Department of Neurology, Landspítali - The National University Hospital of Iceland, Reykjavík, ISL

**Keywords:** small cell lung cancer, paraneoplastic encephalitis, mri brain and spine, acute encephalitis, paraneoplastic syndromes, paraneoplastic neurological syndromes

## Abstract

Paraneoplastic neurological syndrome (PNS) associated with anti-CV2/CRMP5 antibodies is a rare entity that can present in various clinical manifestations, from encephalitis to chorea, depending on the brain region involved. We report a case of an elderly person with small cell lung cancer who presented with PNS encephalitis associated with anti-CV2/CRMP5 antibodies which were confirmed on immunological analysis.

## Introduction

Anti-CV2/collapsin response mediator protein 5 (CRMP5)-related paraneoplastic neurological syndromes (PNS) is a rare form of autoimmune condition that can present with variable neurological manifestations depending on which area of the nervous system is involved, and even in many cases, the paraneoplastic findings precede the tumor [1]. The median survival of these patients can range from 10 to 20 months, but it generally depends on underlying cancer and its related antibodies [2]. We herein present the case of an elderly patient diagnosed with small cell lung cancer (SCLC) and subsequently developed symptoms secondary to the anti-CV2/CRMP5 encephalitis.

## Case presentation

A 70-year-old male patient, a known patient with left lung SCLC with local spread to the mediastinum and pleura being treated with chemotherapy, developed dizziness, decreased power in the legs, sensory changes bilaterally in the feet distally and palms, and constipation. Additionally, the patient also developed gait disturbance. Initially, these symptoms were believed to be related to the chemotherapy; however, to exclude other potential causes, a lumbar puncture was performed, which showed an increase in white blood cells, but the viral panel and gram staining returned negative. However, the immunological panel revealed anti-CV2/CRMP5, anti-Hu-D, and anti-amphiphysin. Electroneuromyography exhibited sensorimotor polyneuropathy of mixed type (axonal/demyelination) with subacute denervation in distal lower limbs. Intravenous immunoglobulin (IVIG) was given, and the patient was admitted to the palliative ward for a change in pain medication.

**Figure 1:**
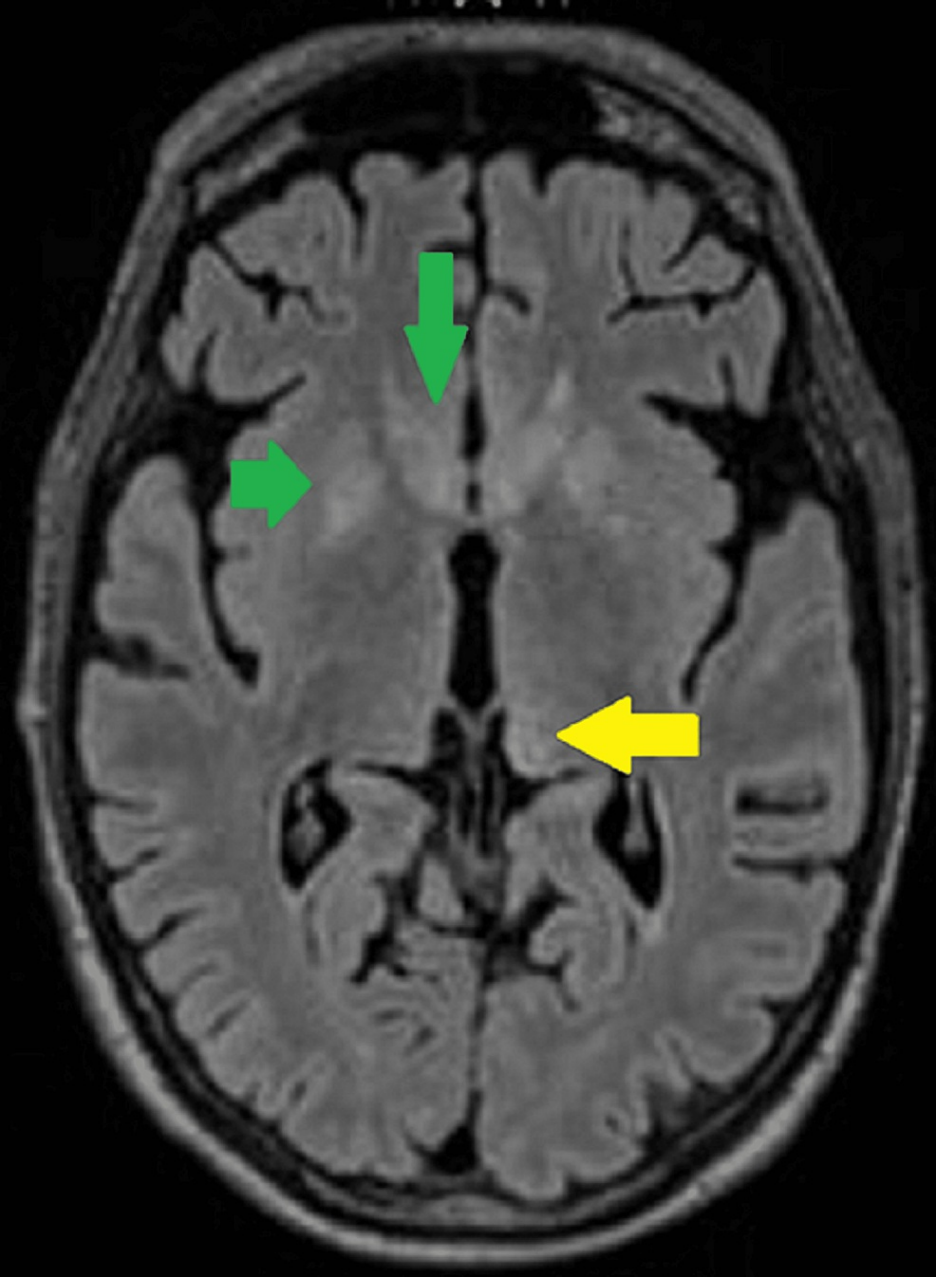
MRI Brain Fluid-attenuated inversion recovery (FLAIR) axial image showing hyperintense signals in the head of bilateral caudate nuclei, putamen (green arrows), and dorsomedially in the left thalamus (yellow arrow).

**Figure 2:**
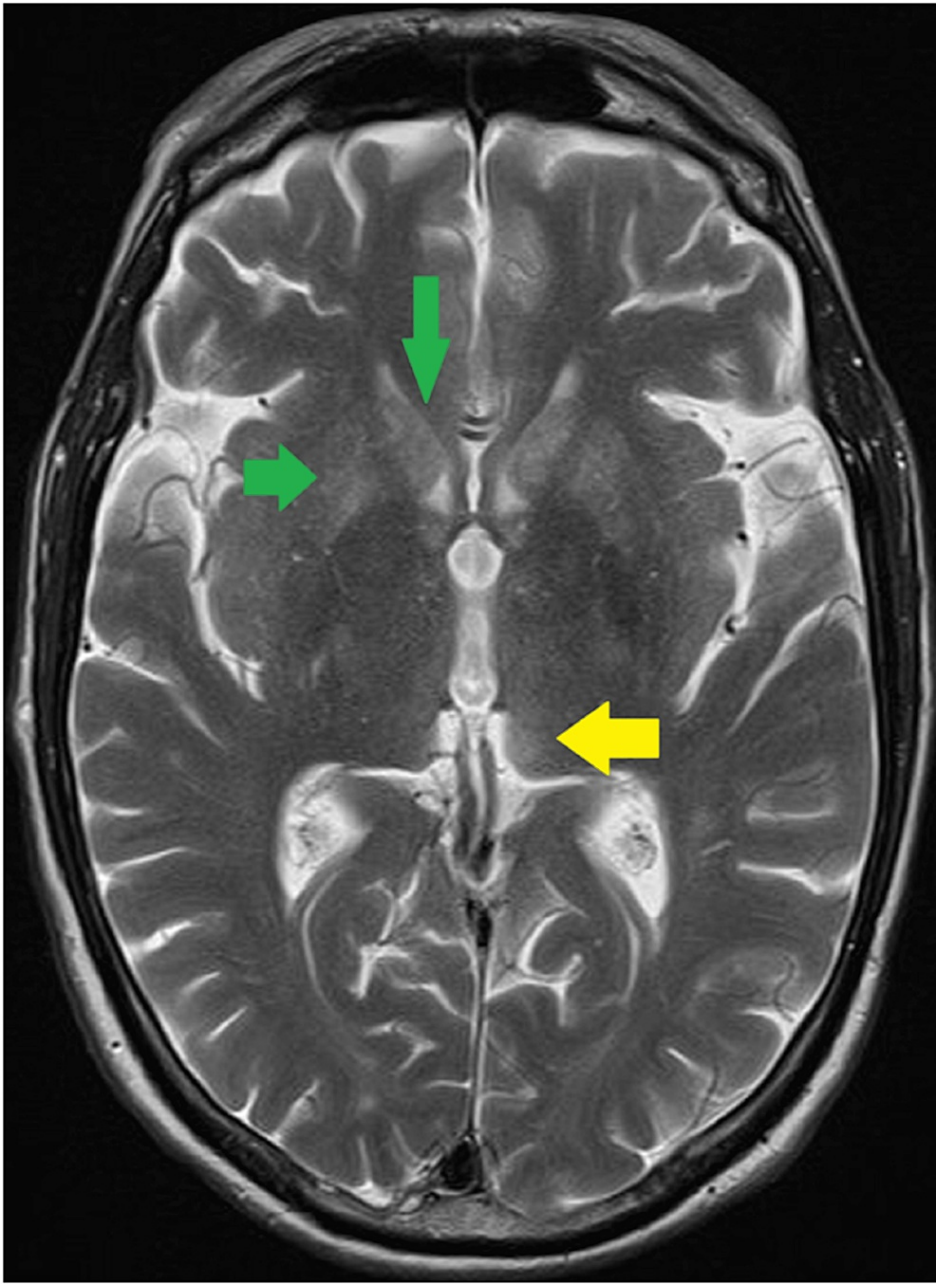
MRI Brain T2-weighted axial image showing hyperintense signals in the head of bilateral caudate nuclei, putamen (green arrows), and dorsomedially in the left thalamus (yellow arrow).

Furthermore, during the admission, the patient also suffered from rapidly increasing neurocognitive symptoms, loss of consciousness, lateral deviation of the right eyeball with a slight change in pupil width, and respiratory failure. Therefore, an urgent brain magnetic resonance imaging (MRI) was performed, which revealed T2 and fluid-attenuated inversion recovery (FLAIR) hyperintense signals in the head of bilateral caudate nuclei, putamen, and dorsomedially in the left thalamus (Figures 1-2). No diffusion restriction or post-contrast enhancement was seen.

In accordance with the patient’s symptoms and localization of changes in the striatum, these changes were suggestive of paraneoplastic anti-CV2/CRMP5 encephalitis. During the admission, the patient also experienced cardiac arrest, which was overturned by cardiopulmonary resuscitation. Pulses of steroids and monoclonal antibodies (rituximab) were also given to the patient with a brief improvement of symptoms. Nonetheless, paraneoplastic symptoms worsened with the onset of delirium and neuropathic pain. Since it was believed that the paraneoplastic symptoms might not be relieved without cancer regression, and the patient’s status was quickly deteriorating, it was decided to proceed with palliative care. Subsequently, the patient passed away several days later.

## Discussion

PNSs are believed to represent an autoimmune response to cancer. This immunological connection is suggested because of the presence of onconeural antibodies in either serum or cerebrospinal fluid and signs of inflammation in the cerebrospinal fluid or MRI in cancer patients with these symptoms. Furthermore, direct and indirect cancer-related disorders, such as infection, metastasis, and nutritional deficits, need to be excluded [3].

Paraneoplastic syndromes are estimated to occur in 8% of patients with cancer. Its prevalence is believed to increase due to the increased survival of cancer patients with improvements in diagnostic methods and therapeutics. Paraneoplastic syndromes result in the formation of onconeural antibodies and associated onconeural antigen-specific T lymphocytes in response to the cross-reactivity between the tumor cells and the nervous system. This is a rare phenomenon and is reported to occur in <1/10,000 of all cancer patients, of which small cell cancer has the highest incidence, up to 5% [4,5]. However, thymoma, colon, renal, and breast cancer can also be associated with PNS [6].

The onconeural antibodies are divided into three main types: 1) well-characterized antibodies with strong cancer association, e.g., anti-CV2/CRMP5, anti-Hu/anti-neuronal nuclear antibody type 1 (ANNA-1), anti-Ri (ANNA-2), anti-Ma2, anti-recoverin, anti-amphiphysin, and anti-Yo/Purkinje cell cytoplasmic antibody type 1 (PCA-1). 2) partially characterized antibodies, e.g., ANNA-3, anti-mGLUR1, and anti-Tr. 3) antibodies that form in both tumor and non-tumor-associated syndromes, e.g., anti-acetylcholine receptor (AchR) and anti-nicotinic AchR [7].

Paraneoplastic anti-CV2/CRMP5 inflammatory disease can present with different symptoms, which depend on the area of the nervous system involved. For example, in anti-CV2 antibody-related encephalitis, patients usually develop choreiform movement disorders, and involvement of the striatum is the prominent radiological feature, as seen in our patient. Anti-Hu antibody-related encephalitis is associated with paraneoplastic encephalomyelitis, cerebellar degeneration, and paraneoplastic subacute sensory neuropathy, and in anti-amphiphysin antibody-related encephalitis patients present with myelopathy, myoclonus and stiff-person syndrome [8].

The diagnosis is usually based on the clinical presentation aided by the radiological findings and confirmed by the laboratory findings with the presence of well-characterized onconeural antibodies. The diagnostic criteria to diagnose “definite PNS” requires the presence of a neurological syndrome, well-characterized onconeural antibodies, and the absence of a brain tumor [9]. Interestingly, in 70-80% of the cases, patients present with symptoms of PNS before the underlying malignancy has been detected [1]. Radiologically, MRI can show T2/FLAIR hyperintense signals in the region involved, and fluorodeoxyglucose-positron emission tomography (FDG-PET) can show hypermetabolic uptake in the involved region [1].

The management of the PNS is challenging, and the treatment options are usually based on expert opinion rather than treatment guidelines due to the lack of randomized controlled clinical trials. The use of IVIGs, methylprednisolone, cyclophosphamide, rituximab, and plasma exchange, in addition to supportive treatment, has been described in the literature [5]. The prognosis of this condition also depends on several factors and is usually poor for patients who develop antibodies against intracellular neuronal antibodies [10].

## Conclusions

Paraneoplastic encephalitis secondary to the anti-CV2/CRMP5 antibodies is an uncommon disease that can occur secondary to SCLC, which should, however, be added to the differential diagnosis in the appropriate clinical setting. The patient’s presentation and prognosis can vary based on the underlying tumor and degree of disease involvement which makes the management of the PNS very challenging.
